# Resonant plasmonic detection of terahertz radiation in field-effect transistors with the graphene channel and the black-As$_x$P$_{1-x}$ gate layer

**DOI:** 10.1038/s41598-023-36802-0

**Published:** 2023-06-14

**Authors:** V. Ryzhii, C. Tang, T. Otsuji, M. Ryzhii, V. Mitin, M. S. Shur

**Affiliations:** 1 Research Institute of Electrical Communication, Tohoku University, Sendai, 980-8577 Japan; 2 Department of Computer Science and Engineering, University of Aizu, Aizu-Wakamatsu, 965-8580 Japan; 3 Department of Electrical Engineering, University at Buffalo, SUNY, Buffalo, NY 14260 USA; 4 Department of Electrical, Computer, and Systems Engineering, Rensselaer Polytechnic Institute, Troy, NY 12180 USA

**Keywords:** Nanoscience and technology, Graphene, Nanophotonics and plasmonics

## Abstract

We propose the terahertz (THz) detectors based on field-effect transistors (FETs) with the graphene channel (GC) and the black-Arsenic (b-As) black-Phosphorus (b-P), or black-Arsenic-Phosphorus (b-As$_x$P$_{1-x}$) gate barrier layer. The operation of the GC-FET detectors is associated with the carrier heating in the GC by the THz electric field resonantly excited by incoming radiation leading to an increase in the rectified current between the channel and the gate over the b-As$_x$P$_{1-x}$ energy barrier layer (BLs). The specific feature of the GC-FETs under consideration is relatively low energy BLs and the possibility to optimize the device characteristics by choosing the barriers containing a necessary number of the b-As$_x$P$_{1-x}$ atomic layers and a proper gate voltage. The excitation of the plasma oscillations in the GC-FETs leads to the resonant reinforcement of the carrier heating and the enhancement of the detector responsivity. The room temperature responsivity can exceed the values of $10^3$ A/W. The speed of the GC-FET detector’s response to the modulated THz radiation is determined by the processes of carrier heating. As shown, the modulation frequency can be in the range of several GHz at room temperatures.

## Introduction

The emergence of the black-Phosphorus (b-P), black-Arsenic (b-As), and the compounds of these materials (b-AsP), with the energy gap $\Delta _{BL}$ varying from 0.15 to 1.2 eV (see, for example,^1–21^) opens new prospects for the creation of different electronic, optoelectronic, and terahertz (THz) devices. The combination of the GLs with the b-As$_x$P$_{1-x}$ layers with graphene^22–28^ can be particularly beneficial for the creation of novel devices, including MIR/FIR/THz interband photodetectors. In this paper, we propose and evaluate the THz detectors akin to the field-effect transistors (FETs) with the graphene channel (GC) and b-As$_x$P$_{1-x}$ gate barrier layer (BL). The operation of such GC-FETs is associated with the carrier heating in the GC by incoming THz radiation (see, for example,^29^) leading to an increase of the thermionic GC-gate current. This implies that the GC-FETs could operate as hot carrier bolometric detectors. The main features of the proposed THz detectors are as follows: (a) the b-As$_x$P$_{1-x}$ BL provides the possibility to choose the desirable BL height (and, hence, optimize the device characteristics) by varying the number of the atomic layers and/or the molar fraction of As^3–6^, (b) the GC exhibits a room temperature elevated carrier energy relaxation time^30–34^, which promotes high detector responsivities and detectivities, and (c) the plasmonic (PL) properties of the GC-FET^35–37^ can enable the detector resonance response to the THz radiation at the frequencies close to the GC frequencies.

**Figure 1:**
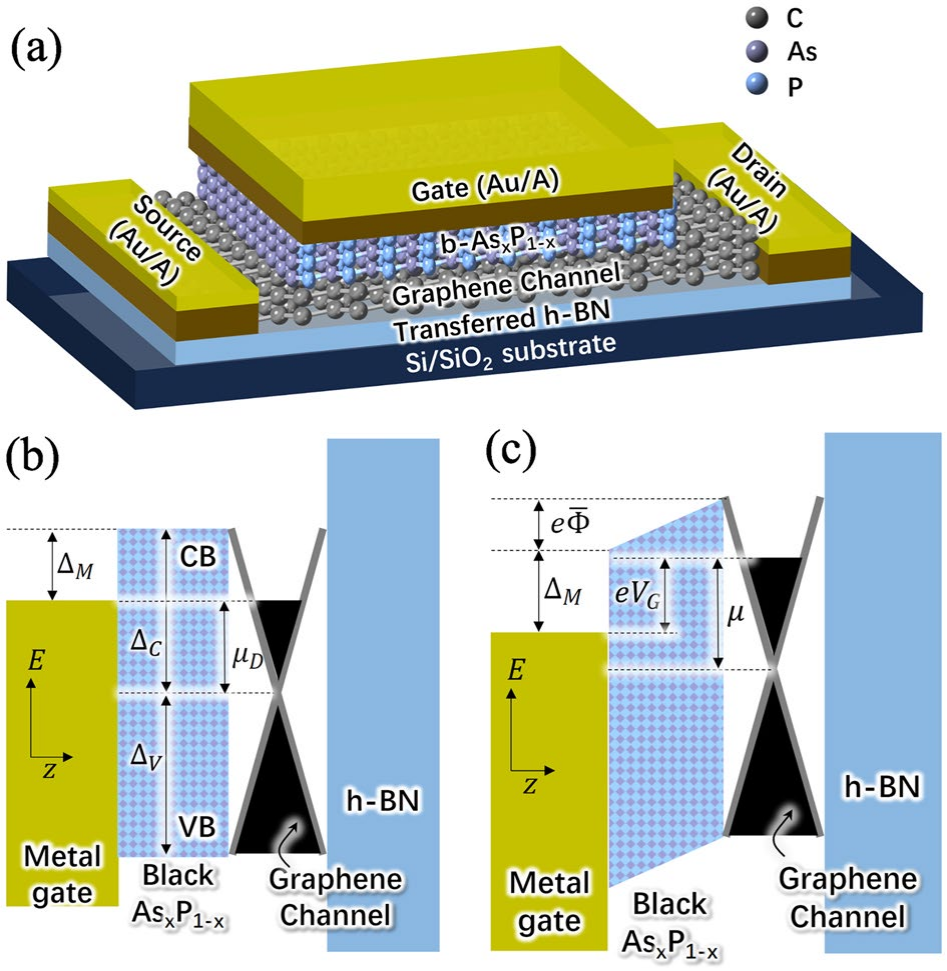
(**a**) Sketch of the GC-FET detector structure and the band diagrams of the GC-FET with $\Delta _C -\mu _D = \Delta _M$ at (**b**) $V_G =0$ (BL flat band condition) and ${{\overline{j}}} = 0$, and (**c**) $V_G > 0$ with $\Delta _C - \mu < \Delta _M$ and $\Delta _V+\mu > \Delta _M$, hence the thermionic electron current density ${{\overline{j}}} > 0$ (the hole current is negligible).

**Table 1:** Metal gate/b-As$_x$P$_{1-x}$/GC barrier layer parameters, $N = 20$, $W = 10$ nm^11–21^.

	*x*	Metal	$\Delta _C$(meV)	$\Delta _V$ (meV)	$\Delta _M$ (meV)	$\mu _D$ (meV)	$\Sigma _D^{=}$ (10$^{12}$cm$^{-2}$)
Sample 1a	0	Al	225	125	85	140	1.60
Sample 2a	1.0	Al	190	90	50	140	1.60
Sample 1b	0	Ti	225	125	105	120	1.17
Sample 2b	1.0	Ti	190	90	70	120	1.17
Sample 1c	0	In	225	125	135	90	0.66
Sample 2c	0.5	In	230	130	130	100	0.81
Sample 1d	0	Mn	225	125	125	100	0.81
Sample 2d	0.5	Mn	230	130	120	110	0.89

## Device structure

Figure 1a shows the GC-FET detector structure (with the number of atomic layers in the BL $N=20$) and the related band diagrams for the assumed band alignment. For definiteness, we consider the GC-FET structures with the n-type GC, in which the thermionic current between the GC and the gate is associated with the electrons overcoming the BL barrier in the conduction band. If $\Delta _V \ge \Delta _C$, where $\Delta _C$ and $\Delta _V$ are the band offsets between the BL conduction and valence band’s edges and the Dirac point in the GC (so that $\Delta _C + \Delta _V$ is the energy gap of the BL), the electron current exceeds the hole current when the electron Fermi energy, $\mu _D$, in the GC is sufficiently large, so that $\Delta _C - \mu _D \lesssim \Delta _M$. Here $\Delta _M$ is the difference between the BL and GC work functions and $\mu _D \simeq \hbar \,v_W\sqrt{\pi \Sigma _D^{=}}$ is the equilibrium value of the electron Fermi energy counted from the Dirac point at $V_G = 0$, $v_W \simeq 10^8$ cm/s is the characteristic electron velocity in GCs, and $\hbar$ is the reduced Planck constant.

Figures 1b and c show the band diagrams for $\Delta _C - \mu _D = \Delta _M$ at zero gate bias ($V_G =0$ with the GC donor density $\Sigma _D^{=}$ corresponding to the BL flat band) and under the gate bias ($V_G > 0$), respectively. We focus on the GC-FETs with n-doped GC, in which the above equality is met, i.e., assuming that in the absence of the bias gate voltage ($V_G = 0$), the BL bottom of the conduction band and the top of the valence band are flat.

Table 1 lists examples of possible combinations of the b-As$_x$P$_{1-x}$ barriers and the metal gate materials. The pertinent parameters were taken from^11–21^.

The results obtained below can be also used for devices with relatively large $\Delta _C$, considering the hole transport instead of the electron one.

The local voltage drop across the BL $e\Phi = \Delta _C - \Delta _M - \mu + e(V_G + \varphi ) = \mu _D-\mu + e(V_G + \varphi )$, where $V_G$ is the applied DC bias gate voltage, $\varphi = \varphi (x,t)$ is the GC potential local value, and $\mu$ is the net electron Fermi energy in the GC. At the GC edges, $\varphi (\pm L, t) = \pm \frac{1}{2}\delta V_{\omega }\exp (-i\omega t)$ or $\varphi (\pm L, t) = \delta V_{\omega }\exp (-i\omega t)$ for the asymmetric (a) and symmetric (s) THz radiation input, respectively, with $\delta V_{\omega }$ and $\omega$ being the amplitude and the frequency of the THz radiation received by an antenna. The asymmetric THz radiation input corresponds to the design when the antenna leads are connected to the GC-FET side (source and drain) contact pads. In the case of the symmetric input, one of the antenna leads is contacted to the gate, whereas the second one to both side contacts.

The variation of the potential difference between the side source and drain contacts (in the case of the symmetrical input) and between the side contacts and the gate leads to the transient electron current along the GC and the transient variation of the self-consistent electron density, i.e., to the excitation of the plasmonic oscillations. In the case of the asymmetric input, the electron current along the GC exists even at very small signal frequency. As a results the electron heating by the incoming signals takes place at such frequencies as well. In contrast, in the case of the symmetrical input, slow variations of the side contacts potential with respect to the gate potential create a very weak lateral electron current not heating the GC electron system. This leads to marked distinctions of the response in the range of low frequencies (which is demonstrated below).

The BL energy gap $\Delta _G$ and the dielectric constant $\kappa _G$ depend on the transverse electric field $\Phi /W$. Accounting for this, one can set $\Delta _C = \eta \Delta _G[1 - (\Phi /WE_G)^2]$, and $\kappa = \kappa _G/[1 - (\Phi /WE_G)^2]$, where $\Delta _G$ and $\kappa$ are the BL energy gap and the dielectric constant in the absence of the transverse electric field, $E_G$ is the characteristic electric field, *W* is the BL thickness (see, for example,^38–41^), and $\eta = \Delta _C/(\Delta _C+\Delta _V)< 1$ the fraction of the BL height related to the conduction band. For the b-P BL with $W = 10$ nm (the number of the atomic layers $N = 20$), $E_G \simeq 0.7 - 0.8\simeq$ V/nm. This implies that the effect of the transverse electric field on $\Delta _C$, $\Delta _G$, and $\kappa _G$ markedly reveals at sufficiently high gate voltages when $\Phi \gtrsim 1$ V). However, such a voltage range is beyond our present consideration. Considering the GC-FETs with a sufficiently thick b-As$_x$P$_{1-x}$ BL at moderate gate voltages, we disregard the carrier tunneling across this layer. This implies that the GC-gate current is associated with the sufficiently energetic electrons overcoming the BL, i.e., it is of thermionic origin.

## Equations of the model

### Thermionic DC and AC

At not-too-small electron densities in GCs, the characteristic time of the electron-electron collisions $\tau _{ee}$ is shorter than the pertinent times associated with the optical phonons $\tau _0$, acoustic phonons $\tau _{ac}$, and impurities $\tau _i$, respectively. This implies that the electron distribution function is close to the Fermi distribution function $f(\varepsilon ) = [\exp (\varepsilon - \mu )/T +1]^{-1}$, characterized by the effective electron temperature *T* generally different from the lattice (thermostat) temperature $T_0$ (in the energy units) and the electron Fermi energy $\mu$. Hence, at $\varepsilon > \mu$, $f(\varepsilon ) \simeq \exp [(\mu - \varepsilon )/T]$. However, in the energy range $\varepsilon > \Delta _C$, the electron escape over the BL can markedly decrease $f(\varepsilon )$. To account for this effect, in the range in question, one can set $f(\varepsilon ) \simeq \xi \exp [(\mu - \varepsilon )/T]$, where $\xi = \tau _{\bot }/(\tau _{ee} + \tau _{\bot })$ with $\tau _{\bot }$ being the electron try-to-escape time.

Considering that the height of the potential barrier for the electrons in the GC and in the metal gate are equal to $\Delta _C - \mu$ and $\Delta _M + e(V_G + \varphi )$, respectively, the density of the thermionic electron current can be presented as

1 $$\begin{aligned} j \simeq j^{max} \biggl [\exp \biggl (\frac{\mu - \Delta _C}{T}\biggl ) - \exp \biggl (-\frac{\Delta _M + e \Phi }{T_0}\biggr )\biggr ]. \end{aligned}$$

Here $j^{max}= e\Sigma /\tau _{\bot }$ is the characteristic (maximum) GC-gate DC density, $\Sigma$ is the electron density in the GC induced by the donors and gate voltage, and $e =|e|$ is the electron charge. One can assume that $\tau _{\bot }$ is determined by the momentum relaxation time, associated with the quasi-elastic scattering of the high-energy electrons, i.e., with acoustic phonons (in sufficiently perfect GCs). Due to this, it is natural to assume that $\tau _{\bot } > \tau _{ac} \gg \tau _{ee}$. The Fermi energy $\mu$ is determined by both the GC doping and the gate voltage.

Equation (1) leads to the following expressions for the thermionic DC density ${{\overline{j}}}$, corresponding to the DC temperature ${\overline{T}}$:

2 $$\begin{aligned} {{\overline{j}}}=j^{max}\biggl [\exp \biggl (\frac{\mu - \Delta _C}{{{\overline{T}}}}\biggl ) -\exp \biggl (\frac{\mu -\Delta _C -eV_G}{T_0}\biggr )\biggr ]. \end{aligned}$$

Due to the dependence of $\mu$ on $V_G$, Eq. (2) provides the GC-gate I-V characteristics. Since ${{\overline{T}}}$ also depends on $V_G$ (because of the electron heating in the GC by the lateral DC), the latter dependence can somewhat contribute to the GC-FET characteristics as well.

At sufficiently high GC lateral conductivity in the situations under consideration (large $\Sigma$ and $\mu$), the DC potential and the DC effective temperature nonuniformity along the GC are weak (${{\overline{T}}}\simeq const$). This implies that we disregard the possible DC crowding. A high electron thermal conductivity additionally suppresses the above nonuniformity.

The AC variation $\delta j_{\omega }$ due to the potential oscillations leading to the electron heating is given by

3 $$\begin{aligned} \delta j_{\omega } = j^{max}\frac{\delta T_{\omega }}{ {{\overline{T}}}} \frac{(\Delta _C -\mu )}{ {{\overline{T}}}}\exp \biggl (\frac{\mu - \Delta _C}{ {{\overline{T}}}}\biggr ) \end{aligned}$$

Here we omitted the term containing the factor $(e\delta \varphi _{\omega }/T_0 )^2/2$ with $\delta \varphi _{\omega }$ being the GC potential ac component. In this case, the quantity $\delta j_{\omega }$, given by Eq. (3), does not depend explicitly on the AC variations of the GC potential (only via the effective temperature variation $\delta T_{\omega }$). This is due to a specific shape of the energy barrier for the electrons in the GC (see Fig. 1c).

### Rectified current and effective carrier temperature

The incoming THz radiation results in variations of the potential in the GC. This leads to extra electron heating and the variation of the electron temperature $\delta T = T - {{\overline{T}}}$. According to Eq. (3), the variation of the net gate current associated with the effect of the incoming THz radiation averaged over its period (rectified photocurrent) is given by

4 $$\begin{aligned}<\overline{\delta J_{\omega }}>= J^{max}{{\mathscr {F}}}(V_G) \frac{<\overline{\delta T_{\omega }}>}{{{\overline{T}}}}. \end{aligned}$$

Here $J^{max} = 2LHj^{max}$, and 2*L* and *H* are the GC length and width,

5 $$\begin{aligned} {{\mathscr {F}}}(V_G) = \frac{(\Delta _C -\mu )}{{{\overline{T}}}}\exp \biggl (\frac{\mu - \Delta _C}{{{\overline{T}}}}\biggr ) = \frac{[\Delta _M -(\mu -\mu _D)]}{{{\overline{T}}}}\exp \biggl [\frac{(\mu -\mu _D) - \Delta _M}{{{\overline{T}}}}\biggr ] \end{aligned}$$

is the barrier factor, and the symbols $<...>$ and $\overline{<...>}$ denote the averaging over the signal period $2\pi /\omega$ and the length of the GC, respectively, with

6 $$\begin{aligned}<\overline{\delta T_{\omega }}>= \frac{1}{2L}\int _{-L}^Ldx<\delta T_{\omega }>. \end{aligned}$$

The dependence of the factor ${{\mathscr {F}}}(V_G)$ on the gate voltage as associated with the voltage dependence of the electron Fermi energy (see below).

The effective electron temperature *T* is determined by the balance of the electron energy transfer to the lattice and the energy provided by the electric field along the GL. At room temperature, the emission and absorption of the optical phonons by the electrons in GLs can be considered as a main mechanism of electron energy relaxation. In this case, the power transferring from the electrons in the GC to the optical phonons due to the intraband transitions is^30–33^.

7 $$\begin{aligned} P_0^{intra} = \hbar \omega _0 R_0^{intra}. \end{aligned}$$

Here

8 $$\begin{aligned} R_0^{intra} = R_0 \frac{\hbar \omega _0\mu ^2}{T_0^3}\biggl [\biggl (1 + \frac{1}{{{\mathscr {N}}}_0}\biggr )\exp \biggl (-\frac{\hbar \omega _0}{T}\biggr )- 1\biggr ], \end{aligned}$$

$\hbar \omega _0 \sim 200$ meV is the optical phonon energy, ${{\mathscr {N}}}_0 = [\exp (\hbar \omega _0/T_0)-1]^{-1} \simeq \exp (-\hbar \omega _0/T_0)$, $R_0$ is the characteristic rate of the interband absorption of optical phonons, and $T_0$ is the lattice temperature. At moderate THz power, the effective electron temperature *T* is close to the optical phonon temperature $T_0$, and Eq. (8) yields for $R_0^{intra}$:

9 $$\begin{aligned} R_0^{intra} \simeq R_0 \frac{\hbar \omega _0\mu ^2}{T_0^3}\biggl (\frac{1}{T_0} - \frac{1}{T}\biggr ). \end{aligned}$$

Equalizing $$ R_0^{intra}$$ given by Eq. (9) and the Joule power associated with the AC in the GC, for the THz range of frequencies (in which one can assume $$\omega \gg 1/\tau _{\varepsilon }$$), we arrive at the following energy balance equation:

10 $$\begin{aligned} \frac{<\overline{\delta T_{\omega }}>}{\tau _{\varepsilon }} = \frac{\textrm{Re}~\sigma _{\omega }}{2\Sigma _0L} \int _{-L}^{L}dx\biggl |\frac{d\varphi _{\omega }}{dx}\biggr |^2. \end{aligned}$$

Here Re $\sigma _{\omega } = \sigma _0\nu ^2/(\nu ^2+\omega ^2)$ is the real part of the GC Drude conductivity, $\sigma _0 = e^2\mu /\pi \hbar ^2\nu$ is its DC value, $\nu$ is the frequency of the electron collisions on impurities, acoustic phonons, as well as due to the carrier viscosity (see^42^, and the references therein). Accounting for the deviation of the optical phonon temperature $T_0$ from the lattice temperature $T_l$, the carrier energy relaxation time $\tau _{\varepsilon }$ associated with the interaction with optical phonons is estimated as^32^ $\tau _{\varepsilon } = \tau _0 (1 + \xi _0)(T_l/\hbar \omega _0)^2\exp (\hbar \omega _0/T_l) \simeq \tau _0 (1 + \xi _0)(T_0/\hbar \omega _0)^2\exp (\hbar \omega _0/T_l)$, where $\tau _0$ is the characteristic time of the spontaneous optical phonon intraband emission by the electrons and $\xi _0 = \tau _0^{decay}/\tau _0$, and $\tau _0^{decay}$ is the decay time of optical phonons in GCs.

### Plasmonic oscillations factor

The description of the spatio-temporal oscillations of the electron density and the self-consistent electric field, i.e., the plasmonic oscillations in the GLs (see, for example^32–37^) forced by the incoming THz signals can be reduced to a differential equation for the AC potential of the gated GC filled by the electrons (followed from a hydrodynamic electron transport model equations^43–45^ coupled with the Poisson equation), $\delta \varphi _{\omega }(x)$ :

11 $$\begin{aligned} \frac{d^2\delta \varphi _{\omega }}{dx^2} + \frac{\omega (\omega +i\nu )}{s^2}\delta \varphi _{\omega } =0, \end{aligned}$$

supplemented by the following boundary conditions:

12 $$\begin{aligned} \delta \varphi _{\omega }|_{x= \pm L}| =\pm \frac{\delta V_{\omega }}{2}\exp (-i\omega t), \qquad \delta \varphi _{\omega }^s|_{x= \pm L}| = \delta V_{\omega }\exp (-i\omega t). \end{aligned}$$

Here $s = \sqrt{4\,e^2\mu \,w/\kappa \hbar ^2}$ is the plasma-wave velocity in the gated GC.

The above equations yield the following formula for the AC potential along the GC

13 $$\begin{aligned} \delta \varphi _{\omega }^a = \frac{\delta V_{\omega }}{2}\frac{\sin (\gamma _{\omega }x/L)}{\sin \gamma _{\omega }}, \qquad \delta \varphi _{\omega }^s = \delta V_{\omega }\frac{\cos (\gamma _{\omega }x/L)}{\cos \gamma _{\omega }}. \end{aligned}$$

Here

14 $$\begin{aligned} \gamma _{\omega } = \pi \frac{\sqrt{\omega (\omega +i\nu )}}{\Omega }, \qquad \Omega = \sqrt{\frac{4\pi ^2\,e^2\mu \,W}{\kappa \hbar ^2L^2}} \end{aligned}$$

are the normalized wavenumber and the characteristic frequency of the plasmonic oscillations of the electron system in the GC-FET under consideration.

The AC electric field along the GC is equal to

15 $$\begin{aligned} \frac{d\varphi _{\omega }^a}{dx} = \frac{\delta V_{\omega }}{2} \frac{\gamma _{\omega }}{L}\frac{\cos (\gamma _{\omega }x/L)}{\sin \gamma _{\omega }}, \qquad \frac{d\varphi _{\omega }^s}{dx} = -\delta V_{\omega } \frac{\gamma _{\omega }}{L}\frac{\sin (\gamma _{\omega }x/L)}{\cos \gamma _{\omega }} \end{aligned}$$

that, accounting for Eq. (12), yields

16 $$\begin{aligned} \frac{<\overline{ \delta T_{\omega }}>^{a,s}}{\tau _{\varepsilon }} = \biggl |\frac{\delta V_{\omega }}{2}\biggr |^2 \frac{\sigma _0}{{\overline{\Sigma }}L^2}{{\mathscr {P}}}_{\omega }^{a,s}. \end{aligned}$$

Here

17 $$\begin{aligned} {{\mathscr {P}}}^a_{\omega } = \frac{\nu ^2}{(\nu ^2+\omega ^2)} \int _0^1d\zeta \biggl |\frac{\gamma _{\omega }\cos (\gamma _{\omega }\zeta )}{\sin \gamma _{\omega }}\biggr |^2, \qquad {{\mathscr {P}}}^S_{\omega } = \frac{\nu ^2}{(\nu ^2+\omega ^2)} \int _0^1d\zeta \biggl |\frac{\gamma _{\omega }\sin (\gamma _{\omega }\zeta )}{\cos \gamma _{\omega }}\biggr |^2 \end{aligned}$$

are the plasmonic factors, which can be also presented as

18 $$\begin{aligned} {{\mathscr {P}}}^a_{\omega } \simeq \biggl (\frac{\pi \nu }{\Omega }\biggr )^2 \frac{\omega }{\sqrt{(\nu ^2+\omega ^2)}}\frac{ P^a_{\omega }}{|\sin \gamma _{\omega }|^2}, \qquad {{\mathscr {P}}}^s_{\omega } \simeq \biggl (\frac{\pi \nu }{\Omega }\biggr )^2 \frac{\omega }{\sqrt{(\nu ^2+\omega ^2)}}\frac{ P^s_{\omega }}{|\cos \gamma _{\omega }|^2}, \end{aligned}$$

with $P^a_{\omega } = \int _0^1d\zeta |\cos (\gamma _{\omega }\zeta )|^2$ and $P^s_{\omega } = \int _0^1d\zeta |\sin (\gamma _{\omega }\zeta )|^2$ being functions of the order of unity oscillating with the frequency. If $\omega \ll \Omega$ ($\gamma _{\omega }$ tends to zero), Eqs. (17) and (18) yield ${{\mathscr {P}}}^a_{\omega } \simeq 1$ and ${{\mathscr {P}}}^s_{\omega } \simeq 0$.

Combining Eqs. (4), (6), and (16), we obtain

19 $$\begin{aligned} \frac{<\overline{\delta J_{\omega }}>^{a,s}}{J^{max}} = \biggl |\frac{\delta V_{\omega }}{2}\biggr |^2\frac{\sigma _0\tau _{\varepsilon }}{{\overline{\Sigma }}L^2} {{\mathscr {F}}}(V_G)\,{{\mathscr {P}}}^{a,s}_{\omega }. \end{aligned}$$

The detector response depends on the antenna type (see, for example,^46,47^). Using an antenna specially desined for the THz range could substantially increase the collected power^47^. Here we define the GC-FET detector current responsivity (in the A/W units) and its voltage responsivity (in the V/W units) as

20 $$\begin{aligned} {{\mathscr {R}}}_{\omega } = \frac{<\overline{\delta J_{\omega }}>^{a,s}}{ S_{\omega }},\qquad {{\mathscr {R}}}_{\omega }^V = \frac{<\overline{\delta J_{\omega }}>^{a,s}}{S_{\omega }} \rho , \end{aligned}$$

respectively. Here $S_{\omega }$ is the THz power collected by an antenna and $\rho = 2L/H\sigma _0$ is the GC DC resistance (for the case of load resistance equal to the GC resistance). This collected power is estimated as $S_{\omega } = I_{\omega }A_{\omega }$, where $I_{\omega }$ is the intensity of the impinging radiation and $A_{\omega } = \lambda _{\omega }^2g/4\pi$ is the antenna aperture^46^, $\lambda _{\omega }$ is the radiation wavelength, and *g* is the antenna gain. Consideringm as an example, the half-wavelength dipole antenna, for which $|\delta V_{\omega }|^2 \simeq I_{\omega } (8\pi /c)(\lambda _{\omega }/\pi )^2$, where *c* is the speed of light in vacuum, we obtain $|\delta V_{\omega }|^2 = 32S_{\omega }/gc$.

Accounting for Eqs. (18) and (19), we obtain

21 $$\begin{aligned} {{\mathscr {R}}}_{\omega } = \frac{32}{g c}\frac{<\overline{\delta J_{\omega }}>^{a,s}}{|\delta V_{\omega }|^2}, \qquad {{\mathscr {R}}}_{\omega }^V = \frac{32}{gc}\frac{<\overline{\delta J_{\omega }}>^{a,s}}{|\delta V_{\omega }|^2}\,\rho . \end{aligned}$$

The latter equations yield

22 $$\begin{aligned} {{\mathscr {R}}}_{\omega }= {{\mathscr {R}}}_0 {{\mathscr {F}}}(V_G){{\mathscr {P}}}^{a,s}_{\omega },\qquad {{\mathscr {R}}}_{\omega }^V = {{\mathscr {R}}_0}^V {{\mathscr {F}}}(V_G)\,{{\mathscr {P}}}_{\omega }^{a,s}, \end{aligned}$$

where

23 $$\begin{aligned} {{\mathscr {R}}}_0 =\frac{16}{g}\frac{e\sigma _0}{{{\overline{T}}} c} \frac{\tau _{\varepsilon }}{\tau _{\bot }}\frac{H}{L},\qquad {{\mathscr {R}}}_0^V= \displaystyle \frac{32}{g}\frac{e}{{{\overline{T}}}c}\frac{\tau _{\varepsilon }}{\tau _{\bot }}. \end{aligned}$$

According to Eq. (23), the characteristic voltage responsivity ${{\mathscr {R}}}_0^V$ does not explicitly depend on the frequency of electron collisions $\nu$.

It is instructive that the responsivity at $V_G=0$ does not turn to zero because of the factor ${{\mathscr {F}}}(0) \ne 0$, so that $<\overline{\delta J_{\omega }} >0$.

## Method and results

**Figure 2:**
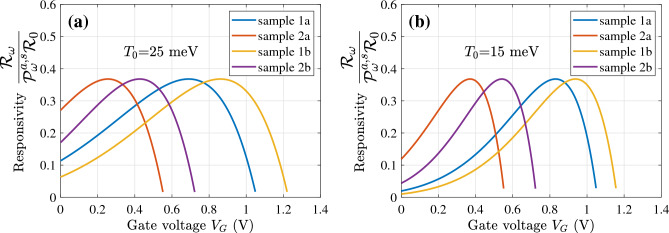
The normalized detector responsivity ${{\mathscr {R}}}_{\omega }/{{\mathscr {P}}}^{a,s}_{\omega }{{\mathscr {R}}}_0$ (the same for the asymmetric and symmetric THz radiation input) as a function of the gate voltage $V_G$ for the GC-FETs with different band parameters: (**a**) at $T_0 = 25$ meV and (**b**) $T_0 = 15$ meV.

**Figure 3:**
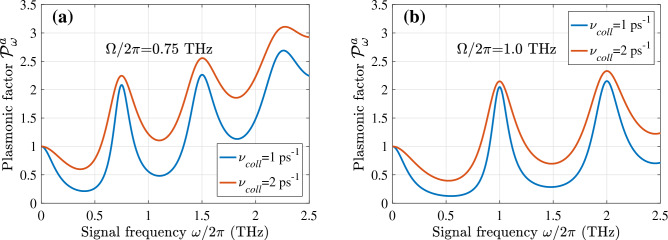
The plasmonic oscillation factor ${{\mathscr {P}}}^a_{\omega }$ of the GC-FETs with the asymmetric THz radiation input versus signal frequency $\omega /2\pi$: (**a**) for $\Omega /2\pi = 0.75$ THz and (**b**) $\Omega /2\pi = 1.0$ THz ($\nu _{coll} = 1$ ps$^{-1}$ and 2 ps$^{-1}$ with $\nu _{visc} = 0.25$ ps$^{-1}$).

**Figure 4:**
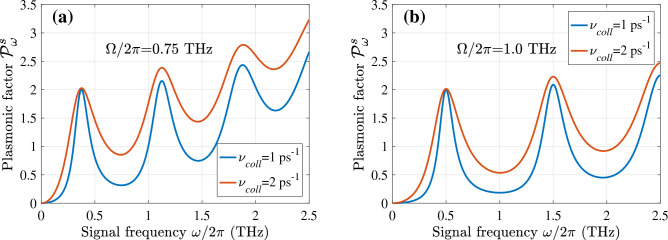
The same as in Fig. 3 but for the plasmonic oscillation factor ${{\mathscr {P}}}^s_{\omega }$ of GC-FETs with the symmetric THz radiation input.

**Figure 5:**
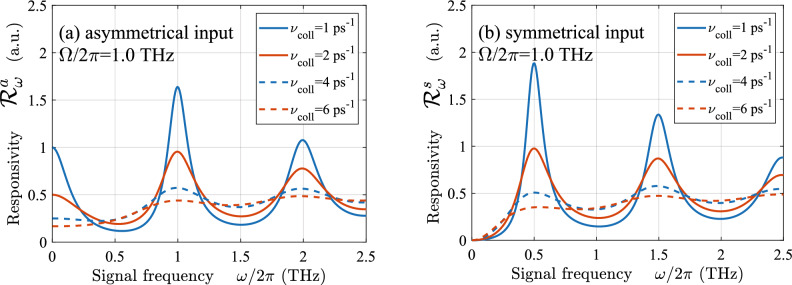
The spectral characteristics of GC-FET detectors current responsivity with (**a**) asymmetric and (**b**) symmetric THz radiation input ($\Omega /2\pi = 1.0$ THz) with different collision frequencies $\nu _{coll}$.

Equations of the model were analyzed analytically and solved numerically. The resulting GC-FET characteristics–their responsivity found for different device samples are demonstrated in Figs. 2, 3, 4, 5.

Figure 2 shows the normalized responsivity at the fundamental plasmonic resonance ${{\mathscr {R}}}_{\omega }/{{\mathscr {P}}}^a_{\omega }{{\mathscr {R}}}_0|_{\omega = \Omega }= {{\mathscr {R}}}_{\omega }/{{\mathscr {P}}}^s_{\omega }{{\mathscr {R}}}_0||_{\omega = \Omega } ={{\mathscr {F}}}(V_G)$ (as a function of the gate voltage $V_G$) for the devices with different $\Delta _C$, $\Delta _V$, $\Delta _M$, and the GC doping corresponding to the BL flat band at $V_G = 0$ calculated using Eqs. (5) and (20). In this case, the thermionic activation energy $\Delta _C - \mu _D = \Delta _V$. Equations (5) and (20) are supplemented by the following relation for $\mu$ accounting for the effect of quantum capacitance^48–51^:

24 $$\begin{aligned} (\mu - \mu _D)(\mu +\mu _D - 2\mu _0) = 2\mu _0eV_G, \end{aligned}$$

where $\mu _0 = (\kappa _G\hbar ^2v_W^2/4e^2W)$. For small (moderate) voltages, Eq. (23) yields

25 $$\begin{aligned} \mu \simeq \mu _D + \frac{\mu _0}{(\mu _D+\mu _0}eV_G. \end{aligned}$$

As seen from Fig. 2, the normalized responsivity, which might be rather high at $V_G = 0$, exhibits a maximum at a certain voltage $V_G^{max}$. The latter is different for different samples depending on the device band parameters. A decrease in the temperature $T_0$ leads to somewhat sharper responsivity versus gate voltage dependence. This is associated with the specifics of the rectified current-voltage dependence given by Eq. (4). The fact that the maximum of function ${{\mathscr {F}}}(V_G)$ height is independent of $T_0$ is reflected in the dependences shown in Fig. 2.

As follows from Eqs. (18) and (22), the maximal values of ${{\mathscr {R}}}_{\omega }$ and ${{\mathscr {R}}}_{\omega }^V$ as functions of the signal frequency $\omega$ are reached at the plasmonic resonances $\omega = \sqrt{n^2\Omega ^2 - \nu ^2} \simeq n\Omega$ for the asymmetrical input, and $\omega = \sqrt{2n-1)^2\Omega ^2/4 - \nu ^2} \simeq (2n-1)\Omega /2$ for the symmetrical input, where $n =1, 2,3,...$ is the plasmonic resonance index. At the fundamental resonances, ${{\mathscr {P}}}^a_{\omega }|_{\omega = \Omega }\simeq 2$ and ${{\mathscr {P}}}^s_{\omega }|_{\omega = \Omega /2} \simeq 1$.

Figures 3 and 4 show the frequency dependence of the plasmonic oscillations factors ${{\mathscr {P}}}^a_{\omega }$ and ${{\mathscr {P}}}^s_{\omega }$ calculated for different values of the plasmonic frequencies $\Omega$ and collision frequencies $\nu$. According to Eq. (22), these factors determine (proportional to) the spectral characteristics of the GC-FET detector responsivity. To account for the electron collisions and the effect of their viscosity on the plasmon damping, we set^42^ $\nu = \nu _{coll} + \nu _{visc}(\omega /\Omega )^2$, assuming $\nu _{coll} = (1 - 2)$ ps$^{-1}$ and $\nu _{visc} = 0.25$ ps$^{-1}$. In the GC-FETs with $L = (0.5 - 1.0)~\mu$m, the latter corresponds to the electron viscosity $h \simeq (250 - 1000)$ cm$^2$/s that is in line with the observed values^42^.

In particular, Figs. 3 and 4 demonstrate that [in line with Eqs. (17) and (22)] the responsivity exhibits fairly sharp (resonant) maxima at $\omega \simeq n \Omega$ and $\omega \simeq (2n-1)\Omega /2$ when $\nu _{coll} = (1 - 2)$ ps$^{-1}$.

Although the GC-FETs with different methods of the THz radiation input exhibit the resonant response, the pattern of the spectral characteristics shown in these plots are rather distinct, and the resonance frequencies differ. This is associated with the excitation of different plasmonic modes (with different spatial distributions of the ac potential) using asymmetric and symmetric input. As seen, the amplitude of the plasmonic factor maxima increases with increasing resonance index despite the strengthening of the viscosity effect. This is attributed to an increase in the average AC electric field when the number of its semi-periods, i.e., the index *n* increase.

Figure 5shows the dependences of the GC-FET detector current responsivity ${{\mathscr {R}}}_{\omega }$ corresponding to the plasmonic factors of Figs. 3b and 4b calculated for $\nu _{coll} = 1$ ps$^{-1}$ and $\nu _{coll} = 2$ ps$^{-1}$ (solid lines). These dependencies exhibit pronounced plasmonic resonances. Since the responsivity ${{\mathscr {R}}}_{\omega }\propto \sigma _0{{\mathscr {P}}}_{\omega } \propto {{\mathscr {P}}}_{\omega }/\nu$, the heights of the responsivity peaks for a larger index *n* and for a larger collisional frequency $\nu$ are smaller. It is instructive that the voltage responsivity ${{\mathscr {R}}}^V_{\omega }$ for different collisional frequencies exhibits different behavior (at the chosen load resistance, which is assumed to be inversely proportional to $\sigma _0$). However, as seen from Fig. 5 (dashed lines), the plasmonic resonances in the cases of much stronger electron scattering ($\nu _{coll} = 4$ ps$^{-1}$ and $\nu _{coll} = 6$ ps$^{-1}$), are substantially smeared.

Using Eq. (14) and setting $\mu =$ 120–140 meV, $\kappa = 4$, $W = 10$ nm, and $L =$ (1–2) $\mu$m, we obtain the following estimate: $\Omega /2\pi \simeq$ (0.53–1.14) THz.

Considering that the escape of a hot electron with the energy $\varepsilon \gtrsim \Delta _C$ from the GC over the BL is possible due to its scattering on an acoustic phonon.

We might assume that the electron escape time $\tau _{\bot } \gg \tau _{ac}$, where $\tau _{ac}$ is the momentum relaxation time for the electrons with the energy $\varepsilon \gtrsim \Delta _C$. The quantity $\tau _{ac}$ can be estimated as^52–54^ $\tau _{ac} \simeq 1$ ps. Considering this, for rough estimates at the values $\Delta _C$ considered above we set $\tau _{\bot } \sim 10 - 20$ ps. The electron energy relaxation time due to the interaction with the GC optical phonons is estimated as $\tau _{\varepsilon } \simeq 32- 65$ ps (compare, for example, with^32,34,55^). The fast decay of optical phonons and the interaction of the electrons in the GC with the interface optical phonons can lead to a decrease in $\tau _{\varepsilon }$ to the values about 10–20 ps. Setting $\mu = 140$ meV, $\nu = 1$ ps$^{-1}$, $\tau _{\bot } = 10$ ps, $\tau _{\varepsilon } = 10$ ps, $g= 1.64$, $H = 2L$, and $(\Delta _C -\mu )/T_0 = 1$, we arrive at ${{\mathscr {R}}}_0 \simeq 4.1\times 10^2$  A/W. This yields the characteristic voltage responsivity ${{\mathscr {R}}}_{0}^V \simeq 3.7\times 10^4$ V/W. The latter values are close to the GC-FET current and voltage responsivities, ${{\mathscr {R}}}_{\omega }|_{\omega =\Omega }$ and ${{\mathscr {R}}}_{\omega }^V|_{\omega =\Omega }$, at the plasmonic resonances.

## Discussion

In Eq. (10), which governs the electron energy balance in the GC, we disregarded the electron cooling effect, associated with thermionic emission. This effect can be accounted for by replacing the quantity $(\tau _{\varepsilon }/\tau _{\bot }){{\mathscr {F}}}(V_G)$ in Eq. (22) by the factor $(\tau _{\varepsilon }/\tau _{\bot }){{\mathscr {F}}}(V_G)/[1+(\tau _{\varepsilon }/\tau _{\bot }){{\mathscr {F}}}(V_G)]$. The pertinent distinction is small if $\tau _{\varepsilon } \lesssim \tau _{\bot }$.

Using the relation between the GC mobility *M* and the Fermi energy $\mu$: $M = ev^2_W/\nu \mu _D$, where the quantity $\mu _D/v_W^2$ is the electron fictitious mass, we find that the values of $\nu _{coll} = (1 - 2)$ ps$^{-1}$ and $\mu _D =(120 -140)$ meV assumed above correspond to $M \simeq (4.3 - 7.1)\times 10^4$ cm$^2$/Vs. For $\nu _{call} = (4 - 6)$ ps$^{-1}$ (see the dashed lines in Fig. 5), one obtains $M \simeq (0.7 - 1.8)\times 10^4$ cm$^2$/Vs, which are realistic GC mobilities at room or somewhat lower temperatures^53,55^. The electron mobility of the GC on b-P studied several years ago^56^, at $T_0 =$(15–25) meV ($T_0 \simeq (180-300)$ K) reaches the values $M\simeq (8-9)\times 10^3$cm$^2$/Vs. This corresponds to $\nu _{coll} \simeq (8--10)$ ps$^{-1}$. Further improvements in the GC/b-P interface quality or/and using the GC remote doping (see, for example,^57,58^), one can reduce $\nu _{coll}$ increasing the plasmonic resonance sharpness. Another option to decrease $\nu _{coll}$ is to use the positively biased back gate, which can electrically induce a sufficient electron density in the GC and, hence, a proper value of the electron Fermi energy, eliminating the necessity of GC doping. The plasmonic resonances and, hence, the pronounced resonant response of the GC-FET detectors might be more pronounced for larger plasma frequency $\Omega$, i.e., in the devices with shorter GCs (smaller length 2*L*). In particular, if $2L = 0.5~\mu$m, the plasma oscillations quality factor is about 8.2 even at $\nu _{coll} = 10$ ps$^{-1}$. One needs to note that even at relatively high values of $\nu _{coll}$, the overdamped plasmonic oscillations can provide elevated GC-FET detector responsivities despite the resonant peaks vanishing.

Electron thermal conductivity along the GC^59^, which leads to the transfer of a portion of the electron heat to the side contacts can reduce the electron temperature and smooth down the spatial nonuniformities of the electron density. The latter can particularly affect the resonant maxima height with increasing plasmonic mode index *n*.

Fairly high values of the GC-FET responsivity are due to the long electron energy relaxation time $\tau _{\varepsilon }$ inherent for GCs. However, the speed of the photodetectors using the hot electron bolometric mechanism is limited by the inverse electron energy relaxation time $\tau _{\varepsilon }^{-1}$ (see, for example,^34,54^). This implies that the operation of the THz GC-FET detectors under consideration (with the parameters used in the above estimates) might be limited to the modulation frequencies in the GHz range.

The GC-FET detector dark current limited detectivity $D^{*}_{\omega } ={{\mathscr {R}}}_{\omega }/\sqrt{2e {{\overline{j}}}}$ (see, for example,^60^) depends, in particular, on the dark current density. As follows from Eqs. (2) and (25), the dark current density is

26 $$\begin{aligned} {{\overline{j}}}\simeq j^{max} \exp \biggl (-\frac{\Delta _M}{T_0}\biggr )\exp \biggl [\frac{\mu _0}{{(\mu _D + \mu _0)}}\frac{eV_G}{T_0}\biggr ]\bigg [1 - \exp \biggl (-\frac{eV_G}{T_0}\biggr )\biggr ]. \end{aligned}$$

For low gate voltages ($eV_G < T_0$), the latter tends to zero as ${{\overline{j}}} \propto V_G$. Since the responsivity in the limit of small $V_G$ is a constant (see Fig. 2), this implies that the GC-FET detector detectivity as a function of the gate voltage increases with decreasing $V_G$ as

27 $$\begin{aligned} D^{*}_{\omega } \propto \frac{1}{\sqrt{V_G}}. \end{aligned}$$

This also means that at low values of $V_G$, the GC-FET noises might be determined by other mechanisms (not by the dark current).

If the electron interactions at the GC/b-AsP interface are relatively strong (leading to high values of $\nu _{coll}$ and preventing the pronounced plasmonic resonance) is a critical issue, the GC-FET structure can be modified by using the length of the gate and the b-AsP layer markedly smaller than the length of the GC 2*L*.

In principle, the GC-FET detectors can be based on the p-type GC, in which $\mu _D <0$. This might exhibit advantages associated with smaller $\Delta _V$ compared to $\Delta _C$ (see Table 1). In the detectors with the p-type GC, a proper thermionic activation energy $\Delta _V +\mu _D$ can be achieved at smaller $\mu _D$, i.e., at the lower carrier (hole) densities. However, the adequate BL and metal gate band alignment might be a problem. This problem can be avoided in the GC-FET structures, in which both the GC and the gate are made of p-type graphene layers (double-GC-FETs). Such double-GC-FETs can exhibit markedly different plasmonic properties. This is because of the possible plasmonic response of the carriers in the double-GG (see, for example,^61–67^). The plasmonic response in the double-GC-FETs depends on the contacts. Depending on the geometry of these contacts, the plasmonic factor can be a fairly different function of the signal frequency. The bolometric detectors based on the double-GC-FET structures with the barrier b-As$_x$P$_{1-x}$ are beyond the scope of our present study and require a separate treatment.

## Conclusions

We proposed and evaluated the THz graphene-channel- FET detectors with the black-Arsenic, black-Phosphorus, or black-Arsenic-Phosphorus barrier gate layers. The operation of these detectors is associated with the hot carrier bolometric effect, i.e., with the carrier heating by incoming THz radiation, causing their thermionic emission from the graphene channel into the gate. Such a THz GC-FET detector can exhibit fairly high characteristics. The excitation of plasmonic oscillations in the graphene channel leads to a strong resonant enhancement of the detector responsivity and detectivity.

The realization of the proposed GC-FET bolometric detectors with elevated characteristics is enabled by the effective carrier heating in graphene accompanied by the effective plasmonic oscillation excitation and the possibility of a proper band alignment between the graphene channel and the barrier layer.

## Data Availability

All data generated or analyzed during this study are included in this published article.
